# Altered Bone Biology in Psoriatic Arthritis

**DOI:** 10.1007/s11926-012-0259-1

**Published:** 2012-05-17

**Authors:** Homaira Rahimi, Christopher T. Ritchlin

**Affiliations:** 1 Department of Pediatrics, Division of Rheumatology, University of Rochester Medical Center, 601 Elmwood Avenue, Box 777, Rochester, NY 14642 USA; 2 Allergy, Immunology, and Rheumatology Unit, University of Rochester Medical Center, 601 Elmwood Avenue, Box 695, Rochester, NY 14642 USA

**Keywords:** Psoriatic arthritis, Ankylosing spondylitis, Osteoclastogenesis, Osteoblastogenesis, New bone formation, Bone biology, Imaging

## Abstract

Psoriatic arthritis (PsA) is characterized by focal bone erosions mediated by osteoclasts at the bone–pannus junction. The bulk of research over the past decade has centered on mechanisms that underlie osteoclastogenesis along with new insights into osteoimmunology; however, recent advances that focus on steps that lead to new bone formation are beginning to emerge. New revelations about bone formation may have direct relevance to PsA given the presence of enthesophytes, syndesmophytes, and bony ankylosis frequently observed in patients with this disorder. In this review, we discuss current developments in the pathogenesis of new bone formation, novel imaging approaches to study bone remodeling and highlight innovative approaches to study the effect of inflammation on bone. Lastly, we discuss promising therapies that target joint inflammation and osteitis with the potential to mediate pathologic bone formation.

## Introduction

The maintenance of bone homeostasis is dependent on a balance between osteoclasts—cells that resorb bone—and osteoblasts—cells that form bone [1]. The past decade of research has yielded critical insights into mechanisms that underlie joint damage, but the most recent studies have unveiled an array of fascinating molecules that modulate signaling pathways of bone formation. With respect to psoriatic arthritis (PsA), long-term consequences of joint inflammation are development of bone erosions alongside new bone formation in the form of syndesmophytes, enthesophytes, and ankylosis (peripheral bony fusion) [2, 3]. Data from Schett and others have further validated differences in the bone pathologies of PsA and rheumatoid arthritis (RA) using new imaging modalities such as micro-CT and have lent credence to the concept of divergent mechanisms of bone repair in these two arthropathies [4]. New insights into cytokine pathways that involve interleukin (IL)-17 and IL-33 may further differentiate mechanisms of bone resorption and repair in PsA and RA and likely will uncover additional therapeutic targets.

Recognition of bone as an active organ that interacts with its environment is a relatively new development [1, 5]. Indeed, the number of molecules with dual roles in bone and immune function is ever-increasing and shows the complexity of the field of osteoimmunology; it may also explain clinical findings such as the notable fragility of bone in patients with chronic autoimmune conditions. Prior hypotheses have suggested use of medications such as steroids to potentially explain increased bone demineralization, but work in osteoimmunology points to specific inflammatory mediators that can target signaling pathways and exert negative effects on bone homeostasis [6–8].

Another central question focuses on pathways of bone formation and includes the view that endochondral bone formation, in which a cartilage template is replaced by bone via the differentiation of chondrocytes into osteoblasts, may be critical in arthritic diseases and is influenced by inflammatory signals that promote osteoblastogenesis [9].

In this paper, we discuss the evolving field of osteoimmunology and how inflammation in PsA and other inflammatory arthritides is leading to new insights into bone remodeling, in particular with regard to new bone formation. We also present recent information gained from novel instruments that image joint and bone inflammation and discuss how current therapies for the treatment of inflammatory arthritis may also affect bone homeostasis.

## Mechanisms of Bone Formation

Chronic joint inflammation is associated with eventual development of bone erosions; however, new bone formation is also observed in patients with inflammatory arthritis, in particular in PsA patients. The development of bony nodules can be seen at sites different from erosions, suggesting an uncoupling of the osteoblast–osteoclast homeostasis that allows for regulated bone turnover and formation [10]. The potential pathways involved in new bone formation include the Wingless (Wnt), transforming growth factor (TGF)-β/bone morphogenetic protein (BMP), and prostaglandin (PG)E_2_ pathways [11].

### Wnt Signaling

The Wnt/B-catenin signaling pathway promotes osteoblast formation by stimulating Runx2, the key transcription factor in activation of genes that mediate osteoblast development. The critical details of osteoblastogenesis are currently under investigation, even as new therapies are in development to target molecules of this pathway. For example, Dickkopf-1 (DKK-1), as a natural antagonist of the Wnt pathway, plays a crucial role in bone remodeling. The impact of anti–DKK-1 antibody on bone formation was analyzed in tumor necrosis factor (TNF)-transgenic mice [12]. Microcomputed tomography (micro-CT) and histopathologic studies demonstrated that bone erosions resolved in joints of mice treated with anti–DKK-1 even in the presence of active joint inflammation. Interestingly, osteophytes developed at the inflamed joints of mice given anti–DKK-1 antibody, a finding not observed in untreated mice. As this murine model typically does not develop osteophytes, this provides preliminary evidence that DKK-1 may act to inhibit local bone formation. The investigators also examined DKK-1 in RA patients, and increased serum levels were observed compared with control patients or patients with ankylosing spondylitis (AS); furthermore, higher levels correlated with increased disease activity.

With regard to new bone formation, a study of AS patients found a correlation between decreased serum levels of DKK-1 and syndesmophyte development when compared with healthy control patients [13], contradictory to prior investigations showing increased DKK-1 serum levels and syndesmophyte formation. The prior methods had measured total DKK-1 levels, whereas Heiland et al. [13] had used an ELISA that measured functional DKK-1 levels as determined by the amount of serum DKK-1 bound to its receptor. The investigators also noted a significant correlation between lower serum levels of DKK-1 and the protein sclerostin, another natural inhibitor of Wnt signaling and negative regulator of bone formation. These findings point to the possible use of DKK-1 and sclerostin as biomarkers of patients at increased risk of development of progressive ankylosis. Given that increased levels of DKK-1 and sclerostin correlate with syndesmophyte formation as outlined above, it is plausible that PsA patients with evidence of new bone formation would also demonstrate elevated levels of these Wnt pathway inhibitors. In contrast, a study by Daoussis et al. [14] that examined levels of DKK-1 in patients with AS, RA, PsA, and controls found elevated DKK-1 levels in patients with AS, but not those with PsA or RA. Clearly, more information is required to elucidate the importance of Wnt signaling and its inhibitors in the process of new bone synthesis in PsA. Certainly the marked heterogeneity of bone phenotypes in PsA complicates interpretation of data and requires a careful assessment of the degree and type of bone pathology (axial, peripheral, erosive, proliferative, or a combination of involvement) in individual patients.

### The TGF-β Superfamily

Another prominent area of study in bone formation has been the TGF-β family, which includes the TGF-β molecules and BMPs [15]. In the canonical TGF-β/BMP pathway, transducer proteins called Smad form a complex through a series of phosphorylation events that leads to translocation of the Smad complex into the nucleus of mesenchymal stem cells to activate bone-specific genes, including the key regulator in bone formation, *Runx2* [16]. The subsequent translocation and gene activation commits the stem cells to osteoblast lineage. Several steps along this path have been studied as potential therapeutic targets, including parathyroid hormone (PTH) and its effects on Smad activation. Additionally, TGF-β is believed to work directly on the PTH receptor to mediate bone remodeling as related to bone formation [17]. A series of experiments by Redlich et al. [18] examined local and systemic bone loss in the TNF transgenic mouse and the effect of anti-TNF therapy combined with bone formation therapy via the use of PTH. They showed osteoblast function is decreased, leading to decreased bone formation, and anti-TNF therapy alone did not result in bone repair. However, combination anti-TNF and PTH led to resolution of bony erosions and new bone formation. When considering AS or PsA patients, it is possible that PTH analogues in combination with anti-TNF therapy could improve or repair bone homeostasis.

### BMP

Recent data suggests that BMPs as a group may be more influential in bone formation than TGF-β. In a study by the Lories et al. [19] laboratory, inhibition of BMP signaling blocked development of ankylosis in a murine model of arthritis, with phenotypic findings similar to AS and PsA, including enthesitis, ankylosis, and dactylitis [19]. In a unique experiment, they inhibited BMP using gene transfer to induce production of noggin, a natural inhibitor of BMP. Through histochemical analysis of joints of mice with and without BMP inhibition, mice transfected with plasmid cDNA plus noggin had decreased ankylosing enthesitis and inhibition of bone formation. Similar histochemistry was seen in human entheses specimens of patients with spondyloarthropathy. In PsA patients, in whom enthesitis and new bone formation are common findings, signaling molecules in the BMP pathway may be a reasonable target for new agents designed to block accumulation of pathologic bone.

Although the pathways in bone formation mentioned above are often studied separately, in reality, there is a complex interplay between them, only some of which has been defined. For example, in a series of experiments using a murine model in which the BMP receptor was knocked out, investigators noted upregulation of Wnt signaling, possibly by targeting Wnt inhibitors DKK-1 and sclerostin [20]. The mice lived to adulthood and had increased bone mass throughout. The findings suggest that studies examining both the BMP and Wnt pathways may provide new insights into synergistic and interactive components in the regulation of bone growth.

### Prostaglandin E_2_

PGE_2_ is a derivative of arachidonic acid that has been acted on by cyclooxygenase (COX) and PGE synthase and is involved in triggering inflammation and pain [21]. The PGE_2_ pathway is also integrally involved in skeletal formation through effects on osteoblast differentiation [22]. Zhang et al. [22] have shown that in COX1 and COX2 knockout mice, bone healing is impaired due to defective osteoblastogenesis. This impairment is resolved with addition of PGE_2_ and BMPs, suggesting a link between the PGE_2_ and BMP pathways. Of the subtypes of PGE_2_ receptors, EP2 and/or EP4 are involved in bone formation and repair [23]. This recent information suggests that NSAIDs may help relieve inflammation and possibly inhibit new bone synthesis in conditions such as AS or PsA. In fact, Wanders et al. [24] demonstrated a greater effect of continuous versus intermittent treatment with celecoxib on inhibition of syndesmophytes in AS, and a recent study showed that NSAIDs plus a TNF antagonist were more effective in the prevention of accumulation of syndesmophytes over time than anti-TNF monotherapy [25]. These studies provide preliminary data to formally address the role of NSAIDs in the prevention of bone fusion in the spondyloarthropathies.

### TNF/RANKL

To understand the relationship of TNF and bone, one must first appreciate the actions of the protein receptor activator of nuclear factor-κB (RANK) [26]. The RANK transmembrane protein is expressed on both osteoclast precursor cells and on fully functional osteoclasts. Upon ligand binding by the RANK ligand (RANKL), the protein acts on adaptor molecule TNF receptor–associated factor (TRAF)-6 to activate nuclear factor-κB (NF-κb), subsequently leading to osteoclastogenesis. The transcription factor activated at the terminus of this pathway is nuclear factor of activated T cells, cytoplasmic 1 (NFATc1). This key regulator of osteoclastogenesis was first noted in T cells and later found to be intimately associated with bone remodeling [27]. However, it remains unclear how the activation of RANK alone triggers intracellular calcium signaling.

Similar to T- and B-cell signaling and the necessity of a secondary signal in order to activate immune pathways, it has been established that activation of osteoclastogenesis requires the presence of a second signal [27]. One possible candidate co-stimulatory pathway involves the triggering receptor expressed in myeloid cells-2 (TREM2)/DAP12/OSCAR molecules. DAP12 is an adaptor molecule found on the surface of cells of myeloid lineage, including osteoclasts, and contains a specialized region known as an ITAM (immunoreceptor tyrosine-based activation motif) necessary for signaling. It is complexed with TREM2 and OSCAR in osteoclasts, leading to a cascade of signaling events that terminate with NFATc1 stimulation by calcium/calcineurin [27]. The ligand of OSCAR was recently suggested to be a neoepitope of fibrillar collagen [28], and the TREM2 ligand remains to be discovered; the source of the ligands for both receptors remains unclear, although work by Koga et al. [29] has suggested that the cells responsible may be osteoblasts. If this is the case, it could provide an explanation for the impairment in both osteoblast and osteoclast function seen in PsA patients and possibly demonstrate why PsA appears to have a different pathogenesis than RA.

### Bruton’s Tyrosine Kinase Inhibition

In elucidating this secondary signal of osteoclastogenesis, one group of molecules being investigated is the Bruton’s tyrosine kinase (Btk) family found on cells with a hematopoietic origin, including B cells, monocytes, and mast cells. Btk is involved in cell proliferation, antibody formation, and release of inflammatory cytokines such as TNF-α, specifically acting in the TREM/DAP12 pathway [30, 31]**.** As similar processes are dysregulated in autoimmune conditions, Btk may play a role in diseases such as inflammatory arthritis. Evidence of this has been seen in murine models of collagen-induced arthritis (CIA), in which arthritis symptoms are ameliorated by Btk small molecule inhibitors. However, given that multiple hematopoietic cell types are involved in CIA, it remains unclear how Btk mediates inflammatory erosive arthritis.

Btk functions in osteoclast differentiation by affecting the stimulation of NFATc1, the known key regulator of osteoclastogenesis [32]. This was found by studying mice with a Btk mutation and noting an impairment of cell–cell fusion (to form multinucleated giant cells) and decreased NFATc1 expression in osteoclast precursors. Additionally, human Btk-deficient osteoclasts from X-linked agammaglobulinemia (XLA) patients are defective at bone resorption activity in vitro, potentially owing to dysregulation of actin cytoskeletal function [33]. These inherent osteoclast defects are corrected by increased inflammatory cytokine levels, which restore osteoclast activity to normalize bone density in these patients. Thus, current evidence points to Btk as a valid candidate co-stimulatory signal in osteoclastogenesis. Studying the Btk pathway is important from the perspective of inflammatory arthritides (e.g., PsA) because inhibition of this molecule may decrease both joint inflammation and osteoclastogenesis.

### Semaphorins

In addition to Btk, another family of molecules just emerging in osteoimmunology is the semaphorins. Semaphorin 4D (Sema4D), originally noted to be an axon guidance molecule [34, 35], has recently been found to have activity in bone remodeling. In a paper by Negishi-Koga et al. [36], Sema4D was shown to be secreted by osteoclasts in order to inhibit osteoblast formation via binding to a cell-surface receptor called plexin-B1, leading to decreased bone formation and overall bone loss. Inhibition of Sema4D in a murine model of osteoporosis led to bone formation by stimulation of osteoblasts and subsequently reversed bone demineralization. It is not known whether inhibition of Sema4D would ameliorate bone loss seen in inflammatory arthritis. As both osteoblast and osteoclast functions are dysregulated in PsA, it is possible that semaphorins may provide the critical clue to understanding the incongruous finding of new bone formation alongside bone erosions.

## Imaging and Bone Pathology

Rheumatology has long used imaging as a means to aid in diagnosis of disease and for measuring outcome after initiation of therapy in PsA; recent work has brought to light other modalities useful for diagnosis at the earliest stages of disease.

### MRI

The use of MRI to evaluate and assess inflammatory arthritic conditions has become routine in rheumatology to minimize exposure to radiation-based modalities. In the inflammatory arthritides, patients with RA have been evaluated and there has been a noted increase in bone edema in this group; however, few studies have looked at MRI in PsA until recently. A study by McQueen et al. [37] looked at the effect of the bisphosphonate zoledronic acid in a small group of PsA patients. Of the six patients on drug versus the six on placebo, they noted that there was no difference in bone erosion scores between the two groups after 1 year. However, there was decreased bone edema, suggesting decreased osteitis as well as decreased disease activity in the treated group. This suggests that bisphosphonates may be useful in PsA patients with regard to ameliorating bone and joint inflammation; however, the lack of effect on resolving erosions may mean that osteoclastogenesis is occurring via a different pathway than in patients with osteoporosis.

### Ultrasound

Interest in the use of ultrasound (US) to assess inflammation has been reignited given the ability of clinicians to perform the examination in the office as well as the cost–benefit analysis compared with other modalities. Furthermore, increasingly sophisticated machines have greatly improved the image quality, and new techniques such as Doppler and contrast have improved the value of the information obtained from US. One recent study looked at PsA patients and compared MRI with contrast enhanced US (CEUS) in diagnosis of early PsA [38], concluding that CEUS is as effective as contrast MRI in detecting early PsA and more effective than noncontrast MRI for early diagnosis. Although there were a small number of patients studied (*n* = 17), the 100 % concordance rate between MRI and CEUS in diagnosing PsA is impressive and points to the efficacy of CEUS in detecting inflammation.

There have been US studies focusing on several aspects of PsA, including bony erosions, axial disease, and swelling [39]. However, a key use of US in PsA is to evaluate enthesitis. Inflammation at the sites of tendon insertion is a key finding in PsA patients, and it can be difficult to assess this by clinical examination or conventional radiography, especially in clinically asymptomatic patients. A unique study by Gutierrez et al. [40] looked entheses sites by power Doppler (PD) US in patients with psoriasis alone and noted 32 % to have evidence of entheseal inflammation, compared with 8 % in a healthy population. These findings suggest that PD US of entheses in PsA patients may be helpful in detecting subclinical disease, which could alter therapeutic management. However, larger studies will be needed to validate these findings and should include a population with early PsA to determine if subclinical etheseal involvement can be detected. Furthermore, similar to MRI, there are limited studies focusing on using US to detect new bone formation in PsA patients.

### Micro-CT

Micro-CT is an imaging modality that can reliably provide high-quality images of bone erosions [41], and a study by Finzel et al. [4] using this instrument noted a novel difference in patients with RA versus PsA. The investigators looked at 30 patients with PsA compared with 58 patients with RA and evaluated the second through fourth metacarphophalangeal joints of the dominantly affected hand. The number of erosions between the two groups was similar, but they noted three distinct types of erosions. Intriguingly, PsA patients had more bottle neck–shaped erosions, with a small cortical break and large erosion beneath and tubule-shaped lesions suggestive of cortical tunnels, although the sizes of the erosions were smaller compared with the RA group. RA patients had U-shaped erosions with wide cortical breaks but smaller erosions beneath and were more likely to be present on the radial side of the joint. The investigators suggested that this difference in location, type, and size of erosion between PsA and RA patients could indicate that there is increased bone formation activity in PsA patients at the cortical surface, leading to smaller erosions and indicating a different repair potential in PsA patients compared with RA. This was further corroborated by the increased number of osteophytes seen in the PsA population.

## Update on Treatment

Because of the increased study of signaling pathways, targeted therapy with biologics and small molecules has greatly expanded the armamentarium of potential agents against inflammation. In the seronegative inflammatory arthropathies, such as PsA and AS, anti-TNF agents have resulted in dramatic changes in the therapeutic approach [42]; however, with regard to effects on bone, studies are still in the early stages to define the precise role of anti-TNF agents and other biologic agents and small molecules in the amelioration of new bone formation. Table 1 describes some of these agents and the possible effects on bone homeostasis.

**Table 1:** Current and potential agents in PsA and effects on bone remodeling

Therapeutic	Target(s)	Molecular structure	FDA indication	Effects on bone
Anti-TNF^a^	TNF, TNF receptor	Antibody or receptor antagonist	RA, PsA, AS, JIA, plaque psoriasis, Crohn’s disease	Inhibits inflammation and osteoclastogenesis, may stimulate osteoblast differentiation [43, 44]
Anti-RANKL^b^	RANKL	Antibody	Certain cancers, bone metastases, osteoporosis	Inhibits osteoclastogenesis [48]
Anti–IL-12/23^c^	Cytokines IL-12 and IL-23	Antibody	Plaque psoriasis	Inhibits inflammation and osteoclastogenesis [56]
Anti–IL-17	IL-17, Th17 cells	Antibody, small molecule against Th17 cells	Phase 1/2 trials	Inhibits inflammation and osteoclastogenesis (via effects on osteoblasts and possibly other cells) [61]
Anti–IL-1^d^	IL-1, IL receptor	Antibody, fusion protein	RA, sJIA, autoinflammatory conditions	Inhibits inflammation and osteoclastogenesis [62]
JAK inhibitors^e^	JAK/STAT pathway	Small molecule	Phase 3 trials	Inhibits inflammation [63]; ? effect on bone
Phosphodiesterase 4 inhibitors^f^	Phosphodiesterase 4	Small molecule	Phase 3 trials	Inhibits osteoclastogenesis (via effects on osteoblasts and possibly other cells) [64]

^a^Adalimumab, infliximab, etanercept

^b^Denosumab

^c^Ustekinumab

^d^Anakinra, canakinumab, rilonacept

^e^Tofacitinib

^f^Apremilast

*AS* ankylosing spondylitis; *FDA* US Food and Drug Administration; *IL* interleukin; *JIA* juvenile idiopathic arthritis; *PsA* psoriatic arthritis; *RA* rheumatoid arthritis; *RANKL* receptor activator of nuclear factor-κB ligand; *sJIA* systemic JIA; *Th17* T-helper type 17 cell; *TNF* tumor necrosis factor

### Monoclonal Antibodies

The TNF antagonists have been the most extensively studied with regard to effects on bone [43, 44]. In PsA, anti-TNF agents decrease synovitis, enthesitis, and dactylitis and retard bony progression [45–47]; however, they do not slow down new bone formation in the axial skeleton, although the effect of these agents on peripheral new bone formation is not known. Overall, these agents likely have an effect on inhibition of osteoclastogenesis via TNF blockade [43]. The ability of these agents to promote healing of bone in PsA has not been evaluated. Another molecule with potent effects on bone is anti-RANKL antibody (denosumab), currently approved for the treatment of osteoporosis [48–50]. This agent blocks bone degradation but can also promote new bone formation, as shown by the increased bone density in osteoporotic patients treated with this agent and in preclinical models. Given that the RANKL pathway in osteoclastogenesis is likely involved in PsA and bone erosion development, this is a potential future therapeutic in this group when combined with an anti-inflammatory agent.

### Small Molecule Inhibitors

Apremilast is a type 4 phosphodiesterase (PDE4) inhibitor that has been recently studied in psoriasis and PsA as well as other inflammatory arthritides [51–53]. It is thought to indirectly block TNF effects due to its inhibition via the cyclic adenosine monophosphate pathway. Because of this, it is believed that any effect on bone is via osteoclastogenesis and TNF blockade. Inhibitors of the JAK family of nonreceptor tyrosine kinases, which are stimulated by various cytokines, have been shown to ameliorate inflammation in murine models of inflammation and in RA [54]; however, the mechanism of inhibition is unclear. No studies to date have evaluated bone homeostasis in patients treated with apremilast or JAK inhibitors, but given the prominent role of inflammatory cytokines on osteoclastogenesis, it is certainly possible that an indirect effect of JAK and PDE4 inhibition is a lower state of bone resorption. In support of the potential bone-protective effects of JAK inhibitors, van der Heijde et al. [55] presented data showing that the oral JAK inhibitor tofacitinib was associated with decreased radiographic progression in RA patients after 1 year of therapy.

### NSAIDs

Use of NSAIDs in seronegative inflammatory arthritis is still an initial treatment modality, especially given numerous studies showing rapid clinical relief of pain and stiffness. However, despite their widespread use, few studies have examined the effect of NSAIDs on bone. Studies looking at NSAIDs in PsA patients are not available, although it would be interesting to see if results would be similar to AS studies suggesting a decrease in radiographic progression [24]. Moving forward, the standard of therapy for axial SpA likely will shift to combination NSAID and TNF inhibition; this therapy may likewise have potential for axial PsA. Similarly to NSAID therapy, disease-modifying antirheumatic drugs are still used as a mainstay in treatment of PsA, but studies looking at effect on bone are lacking.

### Cytokine Research

The inhibition of proinflammatory cytokines (IL-1, TNF, IL-6) has led to many new therapeutic agents that have proven effective and relatively safe. More recently, investigators have studied the IL-17 and IL-23 pathways [56]. A phase 2b, double-blind, randomized, placebo-controlled, crossover study of a monoclonal antibody against the shared p40 subunit of IL-12 and IL-23 in PsA patients met the primary end point of achieving an ACR20 in the treated patients versus control patients [57]. The group also looked at enthesopathy and dactylitis and noted improvement in those findings in the treated group. Based on these data, phase 3 studies were initiated and have recently been completed, but the results are pending. The studies looking at these cytokines did not directly evaluate osteoimmunology, but a study by Sato et al. [58] looking at IL-17 suggests that promotion of osteoclastogenesis may occur indirectly via osteoblasts. The study focused on the subgroup of T cells known as T-helper type 17 (Th17) cells because they secrete IL-17. Based on their experiments, the investigators suggest that IL-17 does not directly affect osteoclasts but rather stimulates osteoblasts to produce RANKL, leading to increased RANK stimulation and subsequent osteoclastogenesis. Added to the story is that IL-17 is believed to be downstream of IL-23; therefore, molecules targeting IL-23 likely would decrease IL-17 expression. In PsA, because it is believed that there is an uncoupling of osteoblast–osteoclast homeostasis, it is possible that the inhibition of IL-17 may not have as profound an effect on osteoclastogenesis as would be expected.

Studies of anti-inflammatory cytokines such as IL-33 [59, 60] suggest this cytokine can inhibit bone resorption via amelioration of the effect of TNF on osteoclastogenesis. Unlike the IL-23/IL-17 pathway, IL-33 therapy may be effective in PsA and bone remodeling because of abrogation of the effects of TNF-α on the RANKL pathway. Further studies of anti-inflammatory cytokines will be necessary to elucidate their effects on bone homeostasis and likely will yield more therapeutic targets.

## Conclusions

**Fig. 1:**
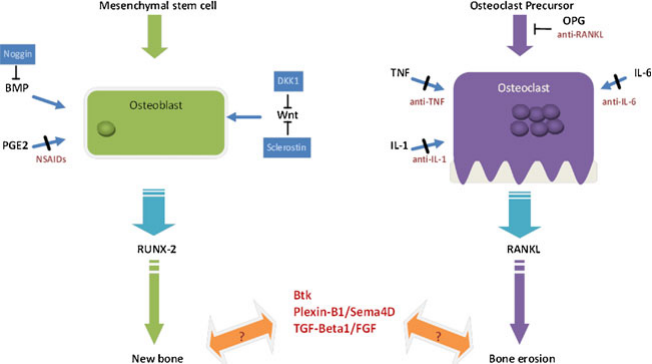
Dual Aspects of Altered Bone Remodeling in Psoriatic Arthritis. In the presence of certain differentiation signals, mesenchymal stem cells are induced to become osteoblasts, bone forming cells. Osteoblasts are then stimulated by via pathways including BMP, PGE2 and Wnt signaling. This leads to activation of the master transcription factor RUNX-2 and subsequent new bone formation. The natural antagonist of BMP is Noggin and of the Wnt pathway is DKK1 and Sclerostin; antibodies to these molecules are currently in development to promote bone formation. In bone resorption, osteoclasts derived from precursors receive signaling from inflammatory molecules such as TNF, IL-1, and IL-6. This activates the master regulator RANKL and subsequent bone erosion. A natural inhibitor of osteoclast formation is osteoprotegrin (OPG). Several therapeutic agents (dark red) are used to block steps in these pathways. Additionally, new molecules and pathways such as Btk, Plexin-B1/Sema4D, and TFG-Beta1 are being studied as their specific roles in bone remodeling have yet to be clarified.

The effect of joint inflammation on bone is of particular interest in PsA, and novel insights into osteoblast function have led to further understanding of bone biology (Fig. 1). It is clear that the signaling pathways such as RANK, Wnt, and BMPs are much more interconnected than previously thought. Similarly, continued research into osteoclastogenesis has helped elucidate pathways involved in bone resorption, leading to the development of new therapeutics, including anti-RANKL agents and anticytokine therapeutics. However, further work needs to be done in order to understand the unique observation in psoriatic patients of simultaneous bone formation and resorption, and suggests that the osteoclast–osteoblast interplay can become uncoupled in the inflammatory microenvironment of the psoriatic joint and enthesis. The use of innovative imaging techniques such as micro-CT and the improved quality of US may help determine how effective the newest biologics and small molecules are in controlling and inhibiting inflammation and subsequent bone and joint damage. Moreover, these instruments may provide important insights into disease pathogenesis. As new insights into osteoimmunology are acquired and applied to what is known about joint disease, we will see the study of osteoblast and osteoclast homeostasis become a pivotal area of focus in PsA.
